# Neonatal Cholestasis: The Changing Etiological Spectrum in Pakistani Children

**DOI:** 10.7759/cureus.25882

**Published:** 2022-06-12

**Authors:** Hazrat Bilal, Muhammad Irshad, Nagina Shahzadi, Almas Hashmi, Hashmat Ullah

**Affiliations:** 1 Pediatric Gastroenterology, Lady Reading Hospital, Peshawar, PAK; 2 Pediatrics, Lady Reading Hospital, Peshawar, PAK; 3 Pediatric Gastroenterology, Children Hospital Faisalabad, Faisalabad, PAK; 4 Pediatric Gastroenterology, Fauji Foundation Hospital, Rawalpindi, PAK; 5 Gastroenetrology, Lady Reading Hospital, Peshawar, PAK

**Keywords:** congenital heart disease, ophthalmologic disorder, metabolic disorder, itching, cholestasis

## Abstract

Objectives: To determine the frequency of clinical presentation and laboratory profile in the diagnosis of the etiological spectrum of neonatal cholestasis.

Material and methods: In this prospective cross-sectional study, we recruited children who presented with jaundice and direct hyperbilirubinemia with onset in the first three months of life. The study was conducted between April 2019 to March 2021 (24 months) at the Government Lady Reading Hospital of Khyber Pakhtunkhwa province in Pakistan. The diagnosis was based on history and clinical findings that included jaundice, stool color, itching, abdominal distention, and deranged liver function tests and confirmed on liver biopsy and specific diagnostic tests. Data was recorded and analyzed using SPSS version 20 (IBM Corp., Armonk, NY).

Results: A total of 90 children were included in the study, out of which 65.6% were male. The average age was recorded as 118.01 days + 118.1 SD. Jaundice, dark urine, and hepatomegaly were found in 85.6% of children while ophthalmologic disorder, congenital heart disease, and itching were the least common symptoms. Laboratory findings of the cholestasis patients showed high bilirubin (mean: 8.88 mg/dL), alanine transaminase (ALT) (mean: 177.48 IU/mL), aspartate transaminase (AST) (mean: 187.11 IU/mL), gamma-glutamyl transpeptidase (GGT) (mean: 187.66 IU/mL) and prolonged international normalized ratio (INR) (mean: 2.20) in majority of patients. The genetic and metabolic disorder was the leading cause found in the majority of children, which was 43.8%.

Conclusion: The common causes of neonatal cholestasis in this study are galactosemia, idiopathic hepatitis, and biliary atresia. The common presentation includes jaundice, hepatomegaly, direct hyperbilirubinemia, raised liver enzymes, and INR.

## Introduction

Neonatal cholestasis is a group of rare diseases with an incidence rate of 1:2500 to 1:5000 [1]. It is caused by a defect in bile synthesis, secretion, or obstruction, resulting in the accumulation of bilirubin, bile salts, and other substances which are excreted through the bile [1]. It is defined as a direct bilirubin level of more than 1 mg/dl when total bilirubin is less than 5 mg/dl or more than 20% when total bilirubin is more than 5 mg/dl in the first three months of life [2,3]. It usually presents with jaundice, clay color stool, dark color urine, itching, failure to thrive, hepatosplenomegaly, and in some cases with congenital malformations such as polysplenia/asplenia, malrotation of the gut, congenital heart diseases, and skeletal anomalies [4]. The most common causes of neonatal cholestasis are biliary atresia and idiopathic neonatal hepatitis. Other causes may be attributed to infections such as sepsis, urinary tract infection, TORCH (toxoplasma, rubella virus, cytomegalovirus, herpes simplex virus, and HIV), metabolic disorders such as galactosemia, tyrosinemia, hereditary fructose intolerance, and progressive familial intrahepatic cholestasis (PFIC), endocrine diseases such as hypopituitarism and hypothyroidism, and congenital malformations of the biliary tract such as choledochal cyst, Caroli disease, and intrahepatic duct paucity [5,6].

Biochemically, it is characterized by an increase in serum direct bilirubin level, raised transaminases, and alkaline phosphatase. Gamma-glutamyl transpeptidase (GGT) is usually raised but may be normal or low [7]. Liver biopsy plays a pivotal role in the diagnosis of various causes of neonatal cholestasis. Common histological findings on liver biopsy are bile duct proliferation, bile plugs, and periportal edema with intact lobular architecture in biliary atresia. Giant cells, mixed inflammatory infiltrate and lobular disarray are present in the case of idiopathic neonatal hepatitis, intracanalicular cholestasis in PFIC while bile duct paucity is characteristic of Alagille syndrome [8]. Early diagnosis and timely management usually result in decreased mortality and long-term morbidity.

## Materials and methods

This descriptive study was conducted between April 1, 2019 to March 31, 2021 at the Department of Child Health, Lady Reading Hospital, Peshawar, Pakistan. All patients who presented with direct hyperbilirubinemia with onset in the first three months of life were included in the study. Written informed consent was taken from parents/guardians (IRB No. 314/LRH/MTI, dated March 25, 2019), after which, demographic data including age, sex, family history, and consanguinity was collected. A detailed history of clinical presentation including jaundice, clay color stool, dark color urine, itching, abdominal distention, and clinical examination of each patient was carried out for jaundice, characteristic facies, hepatomegaly, splenomegaly, ascites, cataract, chorioretinitis, and congenital heart diseases. Biochemical parameters included complete blood count (CBC), bilirubin, alanine transaminase (ALT), aspartate transaminase (AST), GGT, alkaline phosphatase/activate partial thromboplastin clotting time (APTT), albumin, ferritin, triglycerides, urine reducing substances, and urine dipstick for glucose. To determine the etiology, tests of liver biopsy, TORCH serology, thyroid function test, urine for sugar chromatography, urinary succinyl acetone, and bone marrow biopsy were performed. In a few cases, gene mutation analysis was also performed.

Results were compiled and analyzed using SPSS, version 22.0 (IBM Corp., Armonk, NY). Continuous quantitative variables were summarized as mean ± standard deviation (or median and range as appropriate). For categorical variables (ordinal and nominal), frequency and percentages were presented in tabulated form. P-value less than 0.05 was considered significant while the chi-square test was used to check the significance.

## Results

A total of 90 children (65.6% male, 34.4% female) were included in the study, having an average age at presentation of 118.01 ± 118.1 SD; 62.2% of patients had an age of less than three months. The duration of onset of symptoms shows that 62.2% of children have less than five days while 37.8% have more than five days. 

Jaundice was the leading clinical symptom (98.9%) in children that presented with cholestasis followed by dark urine colour (94.4%), and hepatomegaly (85.6%), whereas ophthalmological disorder (2.2%), congenital heart disease (7.8%), ascites (10%), and itching (13.3%) were the least common symptoms (Table 1).

**Table 1:** Clinical symptoms of children with cholestasis (Sample N = 90)

Clinical Symptoms Observed	Count	Percentage
Jaundice	89	98.90%
Color of urine (dark)	85	94.40%
Hepatomegaly	77	85.60%
Consanguinity	63	70.00%
Splenomegaly	42	46.70%
Color of stool (clay)	25	27.80%
Abdominal distension	21	23.30%
Itching	12	13.30%
Ascites	9	10.00%
Congenital Heart Disease	7	7.80%
Ophthalmological Disorder	2	2.20%

Genetic/metabolic disorder was the leading cause found in the majority of children which was 43.8%. PFIC was the leading metabolic disorder (18%) followed by galactosemia (13.5%), and neonatal iron storage disease (7.9%). Idiopathic neonatal hepatitis was found in 25.8%, obstructive cause was 21.3% in which biliary atresia was the leading cause recorded in 13.5% of children while inspissated bile syndrome was found in 4.5% of patients, infection was found in 4.5% and endocrine was noted in 3.4% of patients (Table 2).

**Table 2:** Leading causes of cholestasis amongst sampled children

Causes	Causes Sub-Type	Count	Percentage	Cummulative Percentage
Genetic/Metabolic Disorders	Progressive Familial Intrahepatic Cholestasis (PFIC)	16	18.00%	43.80%
	Galactosemia	12	13.50%	
	Neonatal Iron Storage Disease (NISD)	7	7.90%	
	Niemann Pick Disease (NPD)	2	2.20%	
	Glycogen Storage Disease type 4 (GSD4)	1	1.10%	
	Tyrosinemia	1	1.10%	
Idiopathic Neonatal Hepatitis	Idiopathic Neonatal Hepatitis (INH)	23	25.80%	25.80%
Obstructive	Biliary Atresia	12	13.50%	22.40%
	Inspissated Bile Syndrome	4	4.50%	
	Choledocal Cyst	2	2.20%	
	Caroli's Disease	1	1.10%	
	Spontaneous Perforation of Common Bile Duct	1	1.10%	
Infection	Cytomegalo Virus Hepatopathy	3	3.40%	4.50%
	Urinary Tract Infection	1	1.10%	
Endocrine	Hypothyroidism	3	3.40%	3.40%

Laboratory findings of the cholestasis patients, when stratified over causes, show that the mean bilirubincharacteristicallyhave was high in neonatal iron storage disease (14.78) and galactosemia (10.86). GGT was charateristically high in biliary atresia (289.92) and either normal or low in PFIC (42.75). Similarly, patients with neonatal iron storage disease have high international normalized ratio (INR) value (2.65), and those with biliary atresia, the lowest value (1.72). The highest transaminases were noted in cytomegalovirus hepatopathy and the lowest in neonatal iron storage disease patients (Table 3).

**Table 3:** Stratification of causes - laboratory profile of children with cholestasis ALT: alanine transaminase, AST: aspartate transminase, GGT: gamma-glutamyl transpeptidase, INR: international normalized ratio, INH: idiopathic neonatal hepatitis, BA: biliary atresia, PFIC: progressive familial intrahepatic cholestasis, NISD: neonatal iron storage disease, CMV: cytomegalovirus.

Causes	Direct Bilirubin (mg/dl)		ALT (IU/L)		AST (IU/L)		ALP (IU/L)		GGT (IU/L)		INR	
	Mean	SD	Mean	SD	Mean	SD	Mean	SD	Mean	SD	Mean	SD
BA	6.30	2.09	145.42	138.59	136.91	99.13	1037.92	515.51	289.92	132.32	1.72	0.63
Galactosemia	10.86	5.24	208.00	83.48	287.09	116.44	597.08	466.34	284.92	371.16	2.68	1.75
INH	8.05	5.56	200.39	138.84	198.33	159.21	684.35	481.55	192.61	215.70	1.87	0.97
PFIC	7.94	5.02	221.69	131.19	247.81	156.58	568.81	700.25	42.75	49.35	2.51	1.23
NISD	14.78	8.19	100.00	49.71	113.86	47.36	308.43	202.62	112.57	34.09	2.65	1.48
CMV Hepatopathy	7.53	0.64	341.33	173.75	286.67	178.55	609.00	53.39	129.67	11.55	2.47	1.00

In the case of clinical findings of cholestasis patients, when stratified over causes, results show that clay color stool was significantly high in biliary atresia (55%), PFIC (20%) and idiopathic neonatal hepatitis (15%) as compared to other causes. Similarly, ascites were significantly high in patients with neonatal iron storage disease (50%), galactosemia (33.3%), and PFIC (16.7%). The restthe of parameter were statistically insignificant over the common causes. Itching was also significant in 75% of PFIC, 16.7% with idiopathic neonatal hepatitis, and 8.3% with biliary atresia (Table 4).

**Table 4:** Stratification of causes - clinical profile of children with cholestasis BA: biliary atresia, INH: idiopathic neonatal hepatitis, PFIC: progressive familial intrahepatic cholestasis, NISD: neonatal iron storage disease, CMV: cytomegalovirus.

Symptoms Observed (→)	Itching		Jaundice		Color of stool(clay)		Hepatomegaly		Splenomegaly		Ascites	
	Yes	No	Yes	No	Yes	No	Yes	No	Yes	No	Yes	No
(↓) Causes												
BA	0.00%	19.70%	16.70%	0.00%	55.00%	1.90%	18.20%	0.00%	23.70%	8.60%	0.00%	17.90%
Galactosemia	8.30%	18.00%	16.70%	0.00%	5.00%	20.80%	15.20%	28.60%	15.80%	17.10%	33.30%	14.90%
INH	16.70%	34.40%	30.60%	100.00%	15.00%	37.70%	30.30%	42.90%	21.10%	42.90%	0.00%	34.30%
PFIC	75.00%	11.50%	22.20%	0.00%	20.00%	22.60%	21.20%	28.60%	26.30%	17.10%	16.70%	22.40%
NISD	0.00%	11.50%	9.70%	0.00%	0.00%	13.20%	10.60%	0.00%	5.30%	14.30%	50.00%	6.00%
CMV Hepatopathy	0.00%	4.90%	4.20%	0.00%	5.00%	3.80%	4.50%	0.00%	7.90%	0.00%	0.00%	4.50%
p-value	0		0.82		0		0.623		0.067		0.008	

## Discussion

Neonatal cholestasis is an important group of diseases in the first three months of life. Lack of awareness among health care workers usually leads to late presentation to tertiary care hospitals resulting in high mortality and morbidity [9].

In this study, the average age of presentation was 118.01 days + 118.1 SD which is comparable to a study done by Bazlul Karim et al. who reported the median age of hospitalization as 105.5 days [10]. Metabolic disorders are more common among consanguineous marriages [11]. In our study, the consanguinity was 70.0% which is high as compared to other studies because of the strict tradition of marriages in their tribes. The most common clinical presentations in our study were jaundice in 98.9%, hepatomegaly in 85.6% while clay color stool was found in 27.8%, splenomegaly in 46.7%, and ascites in 10%. These findings were nearly similar to a study done by Dehghani et al. [12] Persistent acholic stool is an important feature of biliary atresia [13]. In this study, 91.7% of patients with biliary atresia had clay color stool as compared to 24.0% of PFIC and 13% of idiopathic neonatal hepatitis with a significant P-value of 0. Itching at presentation is a characteristic feature of PFIC patients. In this study, 93.8% of patients with PFIC had itching as compared to 8.3% of biliary atresia and 8.7% of INH with a significant p-value of 0.

The usual biochemical features of neonatal cholestasis are elevated direct bilirubin, transaminases, and GGT. Among these, GGT is an important marker. High level of GGT coupled with relatively low transaminase are suggestive of biliary atresia. Normal or low levels are then characteristic of PFIC [14,12].

In this study, all patients with PFIC had normal GGT (mean value 42.75 IU/ml). Hepatic transaminases are usually more elevated in infection, infiltrative diseases, and metabolic disorders as compared to biliary atresia. In this study, the mean transaminases (alt/ast) in the PFIC group was 221.6/247.8 IU/ml, in galactosemia, it was 208/287, in cytomegalovirus-hepatopathy, it was 341.3/286.6, and in biliary atresia, it was 145.4/136.9 IU/ml. The most common causes of neonatal cholestasis are biliary atresia followed by idiopathic neonatal hepatitis [15,14]. In Alexandrian and Egyptian studies, idiopathic hepatitis (41.4%) was the most common cause followed by biliary atresia (24.3%) [16].

Our study is unique among studies on neonatal cholestasis because here the most common cause was genetic/metabolic disorders (43.8%) but individually, it was idiopathic neonatal hepatitis (25.8%) followed by PFIC (18%) and biliary atresia (13.5%). These findings were nearly similar to a study done in Sydney in which the most common causes were idiopathic neonatal hepatitis (25%) followed by genetic/metabolic disorders (23%), biliary atresia (20%), and infections (9%) [17].

Liver biopsy plays an important role in the diagnosis of various causes of neonatal cholestasis in the developing world where genetic and enzyme analysis is not easily available. The characteristic features of biliary atresia on liver biopsy are bile duct proliferation, bile plugs, and periportal edema or fibrosis [18,17].

In this study, all patients with biliary atresia had similar findings. Histologically, patients with progressive familial intrahepatic cholestasis present with intracanalicular cholestasis, giant cell transformation, mixed inflammatory infiltrate, and variable degree of fibrosis [19,16]. These findings were consistent with liver biopsy findings in our PFIC patients. All patients with idiopathic neonatal hepatitis showed giant cells, lobular disarray, and mixed inflammatory infiltrate after exclusion of other causes. These findings were similar to other studies.

The main drawback of this study was the lack of genetic analysis in all patients in the genetic and metabolic diseases group, although we did a genetic analysis in 13 patients. Galactose-1-phosphate uridyl transferase (GALT) (c.442C > T [p.Arg148Trp]) mutation was detected in three of galactosemia patients. ABCB11 (c.1708G > A [P.Ala570Thr]) mutation was detected in two, ABCB4 (c.1714C > T [p.Gln572Ter]) in two, and ATP8B1 (c.589-592 > A [p.Gly197Arg]) in three of our PFIC patients, and c.3020C >T [p.Pro1007Leu] mutation was identified in two of our Niemann-Pick type C1 patients. Three neonatal iron storage disease patients in this study showed iron deposition in the epithelium of salivary glands on buccal mucosal biopsy while one tyrosinemia type 1 patient was identified with raised urinary succinyl acetone level.

## Conclusions

The most common causes of neonatal cholestasis in this study were PFIC and galactosemia followed by idiopathic neonatal hepatitis and biliary atresia. The common presentation includes jaundice, hepatomegaly, direct hyperbilirubinemia, raised liver enzymes, and INR. Liver biopsy plays a pivotal role in the diagnosis of cholestatic disorders. Genetic testing is imperative for accurate diagnosis of metabolic and genetic diseases where available.
